# Supplementary material for: Bacterial Tomato Pathogen Ralstonia solanacearum Invasion Modulates Rhizosphere Compounds and Facilitates the Cascade Effect of Fungal Pathogen Fusarium solani
Source: Microorganisms. 2020 May 27;8(6):806. doi: 10.3390/microorganisms8060806 (PMC7356623; doi:10.3390/microorganisms8060806)
Supplement: Supplementary file 1 [file microorganisms-08-00806-s001.pdf]

microorganisms

Supplementary Materials

Bacterial Tomato Pathogen *Ralstonia solanacearum* Invasion Modulates Rhizosphere Compounds and Facilitates the Cascade Effect of Fungal Pathogen *Fusarium solani*

Lv Su [1], Lifan Zhang [1], Duoqian Nie [1], Eiko E. Kuramae [2,3], Biao Shen [1,*] and Qirong Shen [1]

[1] Jiangsu Provincial Key Lab of Solid Organic Waste Utilization, Jiangsu Collaborative Innovation Center of Solid Organic Wastes, Educational Ministry Engineering Center of Resource-saving fertilizers, Nanjing Agricultural University, Nanjing 210095, China
[2] Microbial Ecology Department, Netherlands Institute of Ecology (NIOO-KNAW), Droevendaalsesteeg 10, 6708 PB Wageningen, The Netherlands
[3] Ecology and biodiversity, Institute of Environmental Biology, Utrecht University, Padualaan 8, 3584 CH, Utrecht, The Netherlands
* Correspondence: shenbiao@njau.edu.cn; Tel.: 86-25-8439-6104; Fax: 86-25-8439-6104

Table S1. Primers for PCR and qPCR.

Primer	Sequence(5'-3')	Target gene	Reference
27F	AGAGTTTGATCMTGGCTCAGC	16S rRNA	[1]
1492R	GGTTACCTTGTTACGACTT		
Eub338	ACT CCT ACG GGA GGC AGC AG	16S rRNA	[2]
Eub518	ATT ACC GCG GCT GCT GG		
flicF	GAACGCCAAcGGTGCGAACT	*flic*	[3]
flicR	GGCGGCCTTCAGGGAGGTC		
ITS	TCCGTAGGTGAACCTGCGG	ITS	[4]
5.8S	CGCTGCGTTCTTCATCG		
srf1	GCTTCGTTCACTTCACGGTAGG	*srf*	[5]
srf2	ATGGAGGAAAGACTCGGCTTTT		
fen1	TGGATGGTTCCTCCGCTATCTA	*fen*	[5]
fen2	GGTGACGACCGCGCATTTATT		
itu1	GCCTCCTGCTCATTGTCCTT	*itu*	[6]
itu2	GACGGCGTATCGTGGAGAAT		
bac1	CTGAAGGGACAAGCAGTGAG	*bac*	[5]
bac2	GATAGGAGACGGGTGGGATA		
dfn1	AGTAGTTTTTCTCATCGGTCTC	*dfn*	[5]
dfn2	GGCTCCTTATATTGGGGCATTC		
AFP346	GGTATGTTCACAGGGTTGATG	ITS I	[7]
ITS1-F	CTTGGTCATTTAGAGGAAGTAA		

Table S2. PCR and qPCR conditions for the different primer pairs used in this study.

Primer	Use	Initial denaturing	No. of Cycles	Denaturing	Annealing	Extension	Final extension
27F<br>1492R	PCR	95°C, 5 min	35	95°C, 30 sec	60°C, 30 sec	72°C, 60 sec	72°C, 5 min
Eub338<br>Eub518	qPCR	50°C, 2 min<br>95°C, 2 min	40	95°C, 15 sec	60°C, 60 sec		
flicF<br>flicR	qPCR	95°C, 10 sec	40	95°C, 5 sec	60°C, 30 sec		
ITS<br>5.8S	qPCR	95°C, 30 sec	40	95°C, 5 sec	60°C, 34 sec		
srf1<br>srf2	qPCR	95°C, 10 min	40	95°C, 15 sec	57°C, 60 sec		
fen1<br>fen2	qPCR	95°C, 10 min	40	95°C, 15 sec	57°C, 60 sec		
itu1<br>itu2	qPCR	95°C, 2 min	40	95°C, 15 sec	60°C, 20 sec		
bac1<br>bac2	qPCR	95°C, 10 min	40	95°C, 15 sec	57°C, 60 sec		
dfn1<br>dfn2	qPCR	95°C, 30 sec	40	95°C, 5 sec	60°C, 34 sec		
AFP346<br>ITS1-F	qPCR	95°C, 10 min	45	95°C, 15 sec	60°C, 60 sec		

microorganisms

MDPI

Table S3. Relative abundances of dominant OTUs (top 20) in the rhizosphere of tomato plants.

OUT_ID	Genus	Relative abundance (%)	
		H	D
OTU1	*Fusarium*	8.45	30.04
OTU4	*Thielavia*	31.45	3.06
OTU3	*Plectosphaerella*	2.82	24.82
OTU5	*Chaetomium*	12.68	3.66
OTU12	*Chrysosporium*	6.92	0.49
OTU8	*Podospora*	5.95	0.60
OTU10	*Arthroderma*	1.56	2.56
OTU9	*Ctenomyces*	0.72	3.40
OTU7	*Gibellulopsis*	0.57	3.30
OTU19	*Humicola*	3.02	0.11
OTU13	*Mortierella*	2.97	0.15
OTU11	*Nectria*	0.20	2.81
OTU14	*Mortierella*	2.58	0.42
OTU17	*Ascobolus*	0.02	2.90
OTU15	*Arthroderma*	0.00	2.79
OTU16	*Fusarium*	0.12	2.51
OTU20	*unclassified*	0.07	2.05
OTU37	*Arthroderma*	0.00	2.02
OTU31	*unclassified*	1.31	0.27
OTU22	*Sarocladium*	1.28	0.17

Table S4. Hub taxa of the fungal network of healthy tomato plant rhizospheres.

OUT_ID	Link numbers	Genus
OTU397	19	*Aspergillus*
OTU166	18	*Arthroderma*
OTU341	16	*unclassified*
OTU69	16	*Rhizoctonia*
OTU120	15	*unclassified*
OTU694	15	*Arthroderma*
OTU34	15	*Pseudogymnoascus*
OTU158	14	*unclassified*
OTU140	14	*unclassified*
OTU244	14	*unclassified*
OTU314	14	*unclassified*
OTU4	14	*Thielavia*
OTU185	13	*Ophiocordyceps*
OTU117	13	*Arthroderma*
OTU115	13	*Nigrospora*
OTU386	13	*Arthroderma*
OTU413	13	*Chrysosporium*
OTU100	12	*Didymella*
OTU1281	12	*unclassified*
OTU180	12	*Arthroderma*
OTU511	12	*Penicillium*
OTU3	12	*Plectosphaerella*
OTU167	11	*Panaeolus*

OTU228	11	*Arthroderma*
OTU38	11	*Corynespora*
OTU924	11	*Thielavia*
OTU189	11	*Natantispora*
OTU160	11	*Paecilomyces*
OTU1420	11	*Pseudogymnoascus*
OTU72	11	*unclassified*
OTU191	11	*Bisifusarium*
OTU146	11	*Mortierella*
OTU5	11	*Chaetomium*

Table S5. Hub taxa of the fungal network of bacterial wilt-diseased tomato plant rhizospheres.

OUT_ID	Link numbers	Genus
OTU158	15	*unclassified*
OTU256	13	*Arthroderma*
OTU36	13	*unclassified*
OTU163	12	*Arthroderma*
OTU55	12	*Chaetomium*
OTU122	11	*unclassified*
OTU48	11	*unclassified*
OTU166	11	*Arthroderma*
OTU218	11	*Cephalotrichum*
OTU141	11	*Chrysosporium*

Table S6. Associations between environmental factors and the fungal community.

Environmental Factors	*P*	*R*
p-hydroxybenzoic acid	0.001	0.86
Vanillic acid	0.003	0.41
Ferulic acid	0.001	0.82
pH	0.001	0.57
C	0.001	0.46
N	0.001	0.65

Table S7. Regression associations between soil factors and dominant OTUs (top 20) by the random forest analysis.

OUT_ID	Soil factor	R^2	*P* value of %IncMSE
OTU1_*Fusarium*	Hydroxybenzoic acid	0.50	0.04
OTU1_ *Fusarium*	pH	0.50	0.05
OTU1_ *Fusarium*	C	0.50	0.03
OTU4_unknown	pH	0.95	0.04
OTU3_*Plectosphaerella*	pH	0.27	0.02
OTU3_*Plectosphaerella*	N	0.27	0.04
OTU5_*Chaetomium*	Vanillic acid	0.84	0.05
OTU5_*Chaetomium*	C	0.84	0.01
OTU12_*Chrysosporium*	C	0.57	0.02
OTU9_*Ctenomyces*	Hydroxybenzoic acid	0.47	0.04
OTU19_*Humicola*	N	0.13	0.03
OTU16_*Fusarium*	Vanillic acid	0.26	0.04

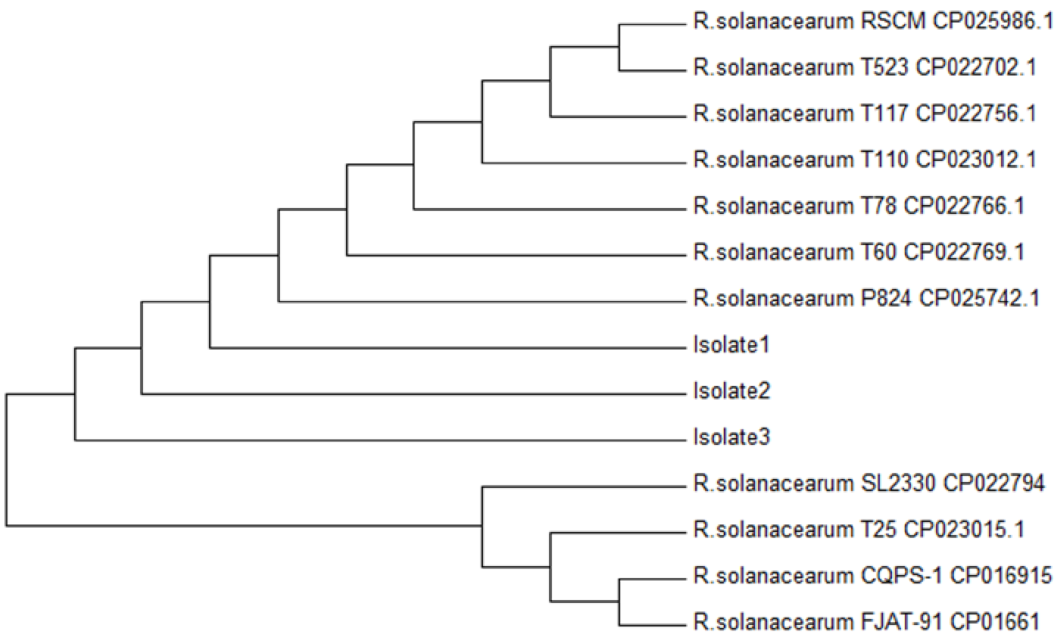

Figure S1. Phylogenetic tree based on 16S rRNA gene sequences of the isolates from bacterial wilt-diseased tomato stems.

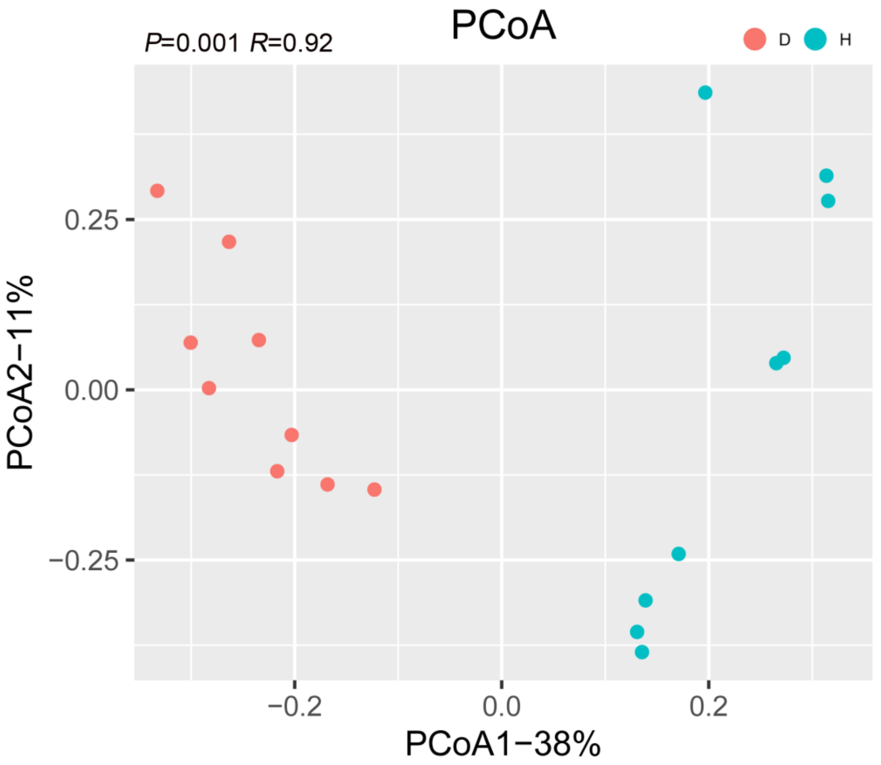

Figure S2. Principal coordinate analysis (PCoA) based on the Jaccard index dissimilarity of presence-absence data in healthy (H) and bacterial wilt-diseased (D) tomato plant rhizosphere fungal communities.

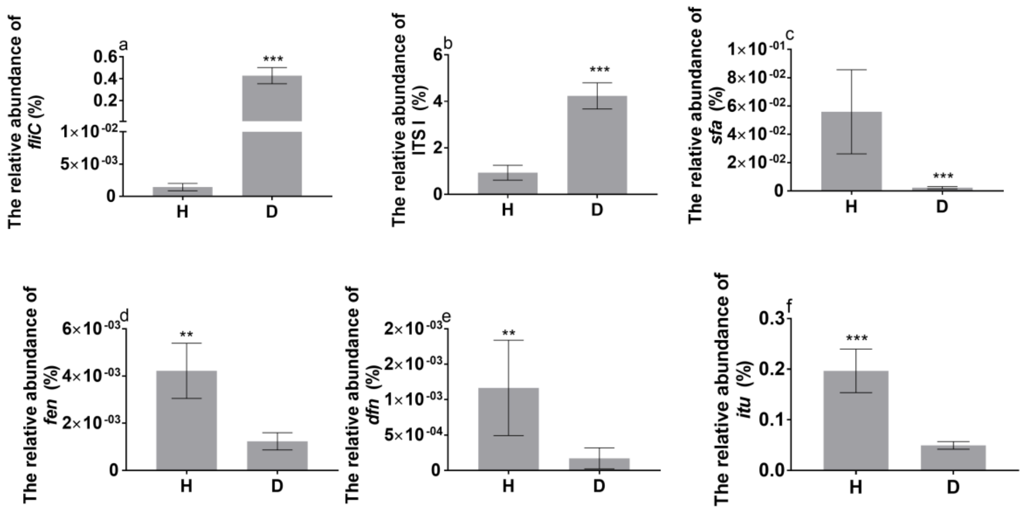

Figure S3. Ratio of functional genes to total bacteria and fungi in healthy (H) and bacterial wilt-diseased (D) tomato plant rhizospheres. *FliC*, ITS I, *sfr*, *fen*, *dfn* and *itu* represent *R. solanacearum*, *F. solani*, sufactin, fengycin, difficidin and iturin, respectively. Statistical significance was determined based on Student's t test. *** $P < 0.001$, ** $P < 0.01$, * $P < 0.05$.

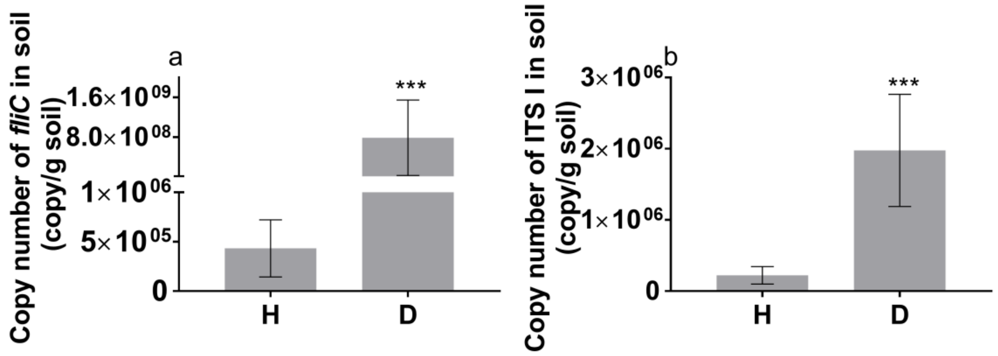

Figure S4. Copy number of *fliC* (a) and ITS I (b) in the rhizosphere of healthy (H) and bacterial wilt-diseased (D) tomato plants in strawberry soil at the florescence stage of tomato plant. Statistical significance was determined based on Student's t test. *** $P < 0.001$.

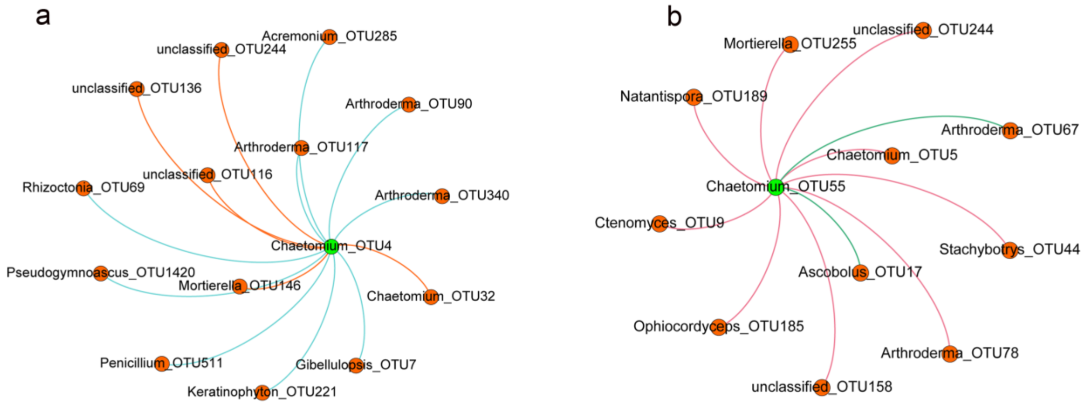

Figure S5. Associations between *Chaetomium* and other OTUs in the fungal network of (a) healthy and (b) bacterial wilt-diseased tomato plant rhizospheres. Orange and blue lines represent negative and positive associations, respectively.

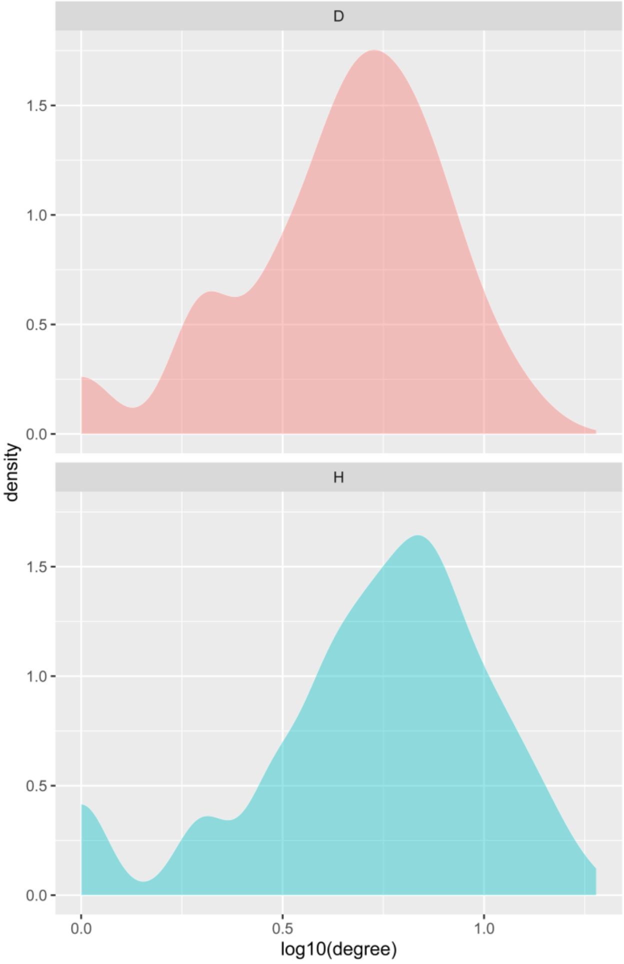

Figure S6. Distribution of the link numbers of the fungal networks of healthy (H) and bacterial wilt-diseased (D) tomato plant rhizospheres.

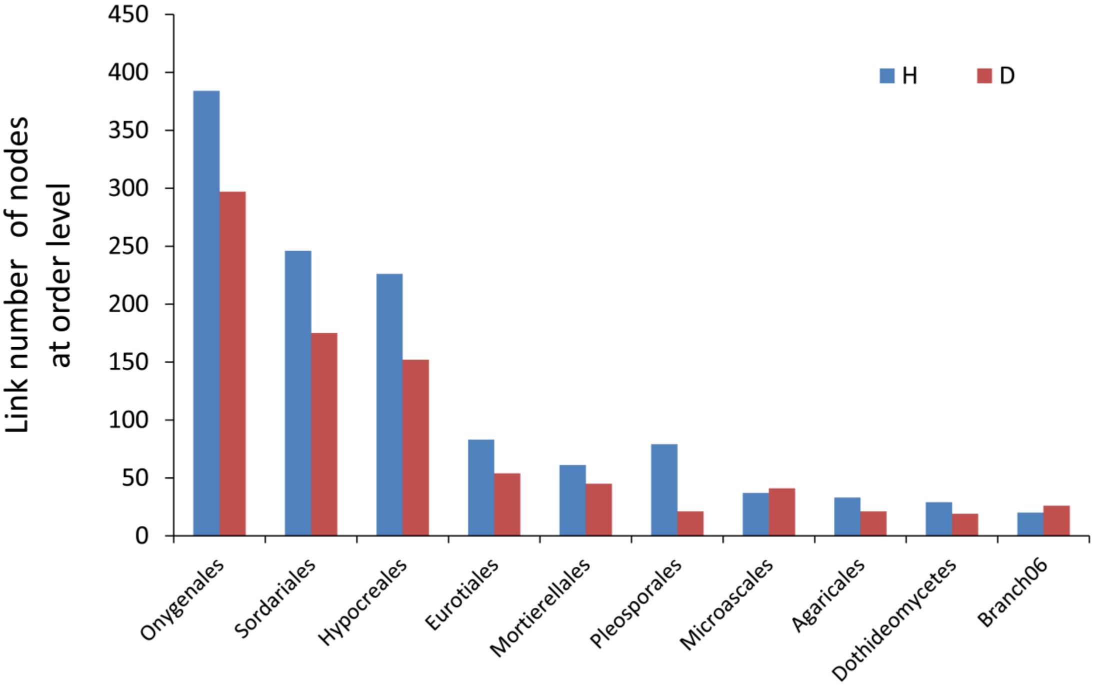

Figure S7. Link numbers of shared orders in the fungal networks of healthy (H) and bacterial wilt-diseased (D) tomato plant rhizospheres.

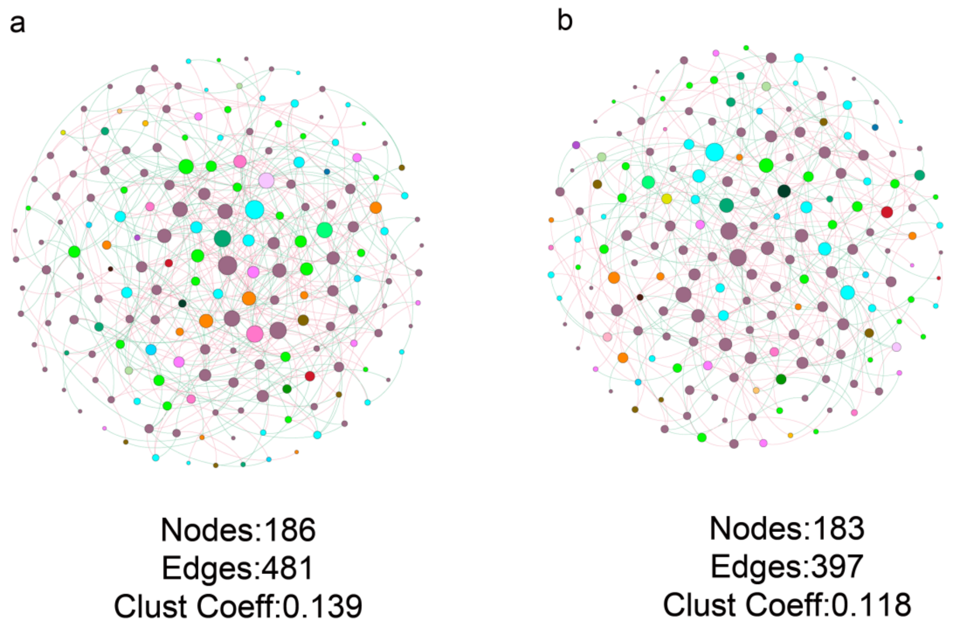

Figure S8. Fungal networks constructed with shared OTUs of (a) healthy and (b) bacterial wilt-diseased tomato plant rhizospheres. The number of nodes and edges and the clustering coefficients are shown below the networks. The node sizes represent link numbers. Yellow and blue lines represent negative and positive associations, respectively. The fungal networks were constructed at the same similarity threshold (0.8).

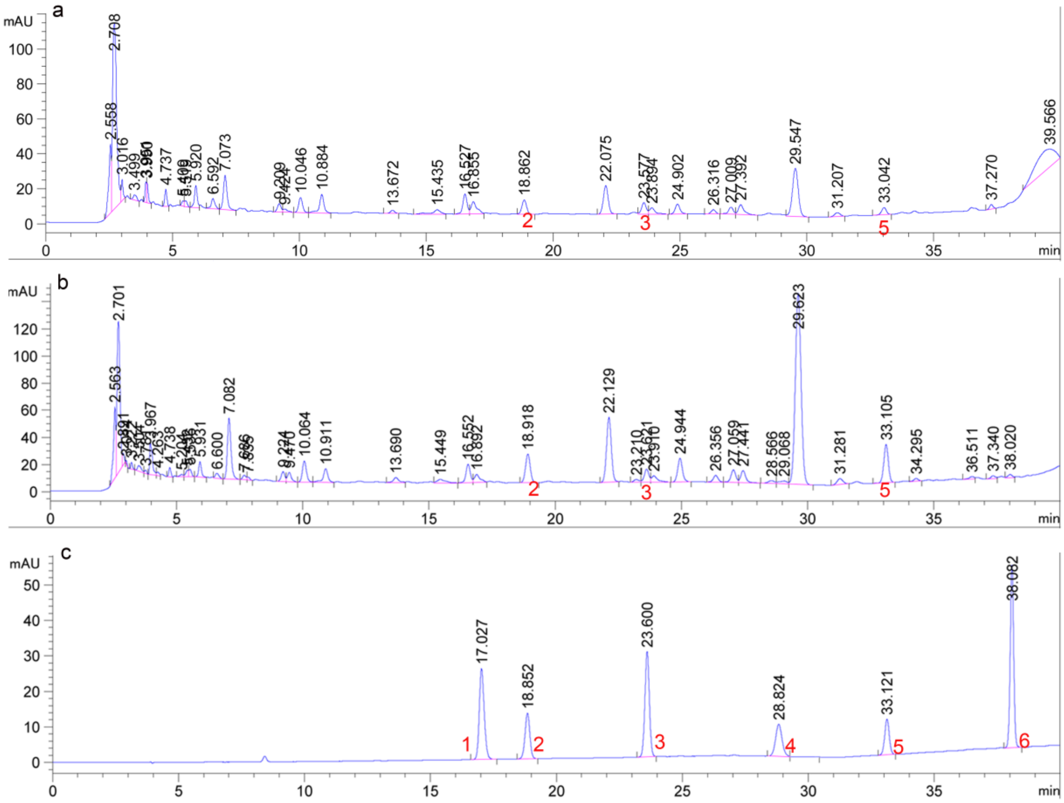

Figure S9. Chromatogram of phenolic acids in rhizosphere soils between healthy (a) and bacterial wilt-diseased (b) tomato plants. 1, Phthalic acid; 2, p-hydroxybenzoic acid; 3, vanillic acid; 4, salicylic acid; 5, ferulic acid; and 6, cinnamic acid.

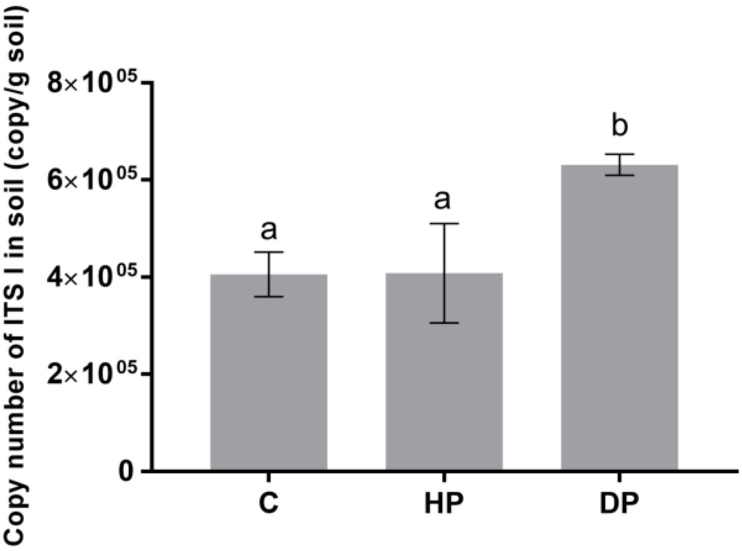

Figure S10. Effect of phenolic acid on the copy number of ITS I (*F. solani*) in soil. C, HP and DP represent the control and treatments with phenolic acid concentrations simulating the healthy and wilt-diseased tomato plant rhizosphere soils, respectively. Error bars indicate one standard deviation from the mean. Different letters indicate significant differences between treatments based on Tukey's test ($P < 0.05$).

References

1. Lalzar, I.; Harrus, S.; Mumcuoglu, K.Y.; Gottlieb, Y. Composition and seasonal variation of Rhipicephalus turanicus and Rhipicephalus sanguineus bacterial communities. *Appl. Environ. Microbiol.* **2012**, *78*, 4110–4116.
2. Le, T-H.; Sivachidambaram, V.; Yi, X.; Li, X.; Zhou, Z. Quantification of polyketide synthase genes in tropical urban soils using real-time PCR. *J. Microbiol. Methods* **2014**, *106*, 135–142.
3. Schönfeld J, Heuer H, Elsas JDv, Smalla K. Specific and Sensitive Detection of Ralstonia solanacearum in Soil on the Basis of PCR Amplification of fliC Fragments. *Appl. Environ. Microbiol* **2003**, *69*, 7248.
4. Pang G, Cai F, Li R, Zhao Z, Li R, Gu X, Shen Q, Chen W. Trichoderma -enriched organic fertilizer can mitigate microbiome degeneration of monocropped soil to maintain better plant growth. *Plant Soil* **2017**, 1–12.
5. Li B, Li Q, Xu Z, Zhang N, Shen Q, Zhang R. Responses of beneficial Bacillus amyloliquefaciens SQR9 to different soilborne fungal pathogens through the alteration of antifungal compounds production. *Front. Microbiol.* **2014**, *5*, 636.
6. Xiong H, Li Y, Cai Y, Yu C, Yan W. Isolation of Bacillus amyloliquefaciens JK6 and identification of its lipopeptides surfactin for suppressing tomato bacterial wilt. *RSC Advances* **2015**, *5*, 82042–82049.
7. Lievens B, Brouwer M, Vanachter ACRC, Cammue BPA, Thomma BPHJ. Real-time PCR for detection and quantification of fungal and oomycete tomato pathogens in plant and soil samples. *Plant Science* **2006**, *171*, 155–165.

© 2020 by the authors. Submitted for possible open access publication under the terms and conditions of the Creative Commons Attribution (CC BY) license (http://creativecommons.org/licenses/by/4.0/).
